# Extracellular matrix remodelling pathway in peripheral blood mononuclear cells from severe COVID-19 patients: an explorative study

**DOI:** 10.3389/fimmu.2024.1379570

**Published:** 2024-06-18

**Authors:** Sarah Louise Murphy, Nora Reka Balzer, Trine Ranheim, Ellen Lund Sagen, Camilla Huse, Vigdis Bjerkeli, Annika E. Michelsen, Ane-Kristine Finbråten, Lars Heggelund, Anne Ma Dyrhol-Riise, Anders Tveita, Aleksander Rygh Holten, Marius Trøseid, Thor Ueland, Thomas Ulas, Pål Aukrust, Andreas Barratt-Due, Bente Halvorsen, Tuva Børresdatter Dahl

**Affiliations:** ^1^ Research Institute of Internal Medicine, Oslo University Hospital Rikshospitalet, Oslo, Norway; ^2^ Institute of Clinical Medicine, Faculty of Medicine, University of Oslo, Oslo, Norway; ^3^ Genomics and Immunoregulation, Life & Medical Sciences (LIMES) Institute, University of Bonn, Bonn, Germany; ^4^ Systems Medicine, German Center for Neurodegenerative Diseases (DZNE), Bonn, Germany; ^5^ PRECISE Platform for Single Cell Genomics and Epigenomics, German Center for Neurodegenerative Diseases and the University of Bonn, Bonn, Germany; ^6^ Department of Medicine, Division of Cardiovascular Medicine, Brigham and Women’s Hospital and Harvard Medical School, Boston, MA, United States; ^7^ Department of Internal Medicine, Lovisenberg Diakonal Hospital, Oslo, Norway; ^8^ Department of Internal Medicine, Drammen Hospital, Vestre Viken Hospital Trust, Drammen, Norway; ^9^ Department of Clinical Science, Faculty of Medicine, University of Bergen, Bergen, Norway; ^10^ Department of Infectious Diseases, Oslo University Hospital Ullevål, Oslo, Norway; ^11^ Department of Internal Medicine, Bærum Hospital, Vestre Viken Hospital Trust, Gjettum, Norway; ^12^ Division of Laboratory Medicine, Department of Immunology, Oslo University Hospital, Oslo, Norway; ^13^ Department of Acute Medicine, Oslo University Hospital, Oslo, Norway; ^14^ Section of Clinical Immunology and Infectious Diseases, Oslo University Hospital Rikshospitalet, Oslo, Norway; ^15^ Thrombosis Research Center (TREC), Division of Internal Medicine, University Hospital of North Norway, Tromsø, Norway; ^16^ Department of Anesthesia and Intensive Care Medicine, Oslo University Hospital, Oslo, Norway

**Keywords:** extracellular matrix, immune response, SARS-CoV-2, COVID-19, lung pathology

## Abstract

There is a reciprocal relationship between extracellular matrix (ECM) remodelling and inflammation that could be operating in the progression of severe COVID-19. To explore the immune-driven ECM remodelling in COVID-19, we in this explorative study analysed these interactions in hospitalised COVID-19 patients. RNA sequencing and flow analysis were performed on peripheral blood mononuclear cells. Inflammatory mediators in plasma were measured by ELISA and MSD, and clinical information from hospitalised COVID-19 patients (N=15) at admission was included in the analysis. Further, we reanalysed two publicly available datasets: (1) lung tissue RNA-sequencing dataset (N=5) and (2) proteomics dataset from PBCM. ECM remodelling pathways were enriched in PBMC from COVID-19 patients compared to healthy controls. Patients treated at the intensive care unit (ICU) expressed distinct ECM remodelling gene profiles compared to patients in the hospital ward. Several markers were strongly correlated to immune cell subsets, and the dysregulation in the ICU patients was positively associated with plasma levels of inflammatory cytokines and negatively associated with B-cell activating factors. Finally, our analysis of publicly accessible datasets revealed (i) an augmented ECM remodelling signature in inflamed lung tissue compared to non-inflamed tissue and (ii) proteomics analysis of PBMC from severe COVID-19 patients demonstrated an up-regulation in an ECM remodelling pathway. Our results may suggest the presence of an interaction between ECM remodelling, inflammation, and immune cells, potentially initiating or perpetuating pulmonary pathology in severe COVID-19.

## 1 Introduction

SARS-CoV-2 infection causes a spectrum of disease manifestations, but with the lungs as its primary target (1), resulting in a spectrum of symptoms ranging from mild respiratory issues to Acute Respiratory Distress Syndrome (ARDS), which is associated with high mortality (2, 3). Emerging evidence suggests that the mechanisms underlying severe COVID-19 involve dysregulation of the immune system, persistent activation of inflammatory pathways as a significant pathogenic mediator (4, 5), and a marked dysregulation of extracellular matrix (ECM) remodelling (6). There is a bidirectional interaction between inflammation and ECM regulation (7, 8). This intricate interplay may also form a pathogenic loop in the progression of severe COVID-19 (9, 10). Thus, in addition to its structural role, the ECM contributes to cell signalling and tissue homeostasis (11). Moreover, degraded ECM fragments can act as signalling molecules, further perpetuating the inflammatory response and exacerbating tissue damage (10, 12). However, the role of ECM remodelling and its interplay with immune cells is still not fully understood in the pathogenesis of COVID-19.

Remodeling of the ECM is orchestrated by various ECM regulatory proteins, including several matrix metalloproteinases (MMPs) and their endogenous inhibitors, tissue inhibitors of metalloproteinases (TIMPs) (7, 13). Disruption of this delicate balance of ECM synthesis and degradation has profound implications for tissue integrity and function. Several studies have reported increased systemic levels of ECM remodelling proteins in COVID-19 (9, 10, 14). The immune responses in peripheral blood during COVID-19 infection have been extensively investigated, including single-cell analyses of peripheral blood mononuclear cells (PBMC) showing dysregulation of several immune-related pathways in relation to disease severity such as a diminished IFNα response in the B cell compartments of individuals with critical and severe disease (15) and altered JAK/STAT, MAPK/mTOR, and nuclear factor κB (NF-κB) signalling (16). Such analyses have also suggested that attenuated IFN-responsive genes and their related signalling pathways could be critical for the progression of pulmonary fibrosis in COVID-19 patients (17). There are also numerous studies on the role of ECM remodelling in COVID-19 (6, 9, 18).

To further investigate the interaction between inflammation and ECM remodelling, we performed transcriptome analyses of mediators of ECM remodelling in COVID-19 patients and examined their relation to inflammatory mediators. Based on published sequences from lung biopsies of deceased COVID-19 patients and proteome analyses of PBMC from hospitalised COVID-19 patients. We examined mediators of ECM remodelling in inflamed and non-inflamed lung tissue in severe COVID-19-related pulmonary disease as well as if the transcriptome profile in PBMC concerning disease severity was reflected at the protein level.

In this explorative study we investigated ECM remodelling mediators in inflamed compared to non-inflamed lung biopsies of deceased COVID-19 patients and PBMC proteome analyses from hospitalised patients. We aimed to understand the relationship between ECM remodelling in severe COVID-19-related pulmonary disease and the translation of PBMC transcriptome profiles to the protein level.

## 2 Materials and methods

### 2.1 Study design and patient population

This study included PBMC from 15 hospitalised COVID-19 patients (≥18 years old) from the original NOR-Solidarity trial (19). Patients were included between March 28 and October 5, 2020. Hence, the collected study period spanned the initial wave of the COVID-19 pandemic in Norway. In the NOR-Solidarity trial, a total of 162 patients were randomised and allocated to one of three treatment arms: local standard of care (SoC), SoC plus oral hydroxychloroquine or SoC plus intravenous remdesivir (19). PBMC were collected from a subgroup of COVID-19 patients at hospital admission before any treatment was administrated. We also included PBMC samples from six age- and gender-matched self-reported healthy controls for comparison. Demographics for patients and healthy controls included in this study are shown in **Table 1**. For more detail on population demographics, see **Supplementary Table S1**. To demonstrate the representativeness of patients in this sub-study, the demographics of this subpopulation are also presented together with the demographics of the whole NOR-Solidarity study population in **Supplementary Table S1**.

**Table 1:** Demographic, clinical, and biochemical characteristics of the 15 COVID-19 patients included in the original NOR-Solidarity cohort sent to RNA sequencing, and age-matched healthy controls (HC).

Parameter	COVID-19 patients n=15	HC n=6
Age, years	60.0 ± 13.4	56.7 ± 13.0
Male gender (%)	12 (80)	3 (50)
Body Mass Index, kg/m2	29.5 ± 4.7	NA
Vaccinated ≥ 1 dose (%)	0 (0)	0 (0)
Oxygen therapy (%)	11 (73)	0 (0)
ICU admission (%)	5 (33)	0 (0)
P/F-ratio at admission, kPa	39.6 (30.4, 45.4)	NA
Haemoglobin, g/dL	13.6 ± 1.1	11.7–15.3^a^
C-reactive protein, mg/L	85 (48, 161)	<4^a^
Ferritin, µg/L	586 (293, 1065)	10–170^a^
White blood cell count, x 10^9^/L	7.8 ± 4.0	3.5–10^a^
Neutrophils, x 10^9^/L	6.3 ± 4.1	1.5–7.3^a^
Lymphocytes, x 10^9^/L	1.0 ± 0.4	1.1–3.3^a^
eGFR (ml/min/1.73 m^2^)	93.3 ± 25.9	<60^a^

ICU, intensive care unit, P/F Ratio; PaO2/FiO2-ratio; eGFR, estimated glomerular filtration rate; HC, healthy controls; NA, Not available. Values are presented as mean ± SD or median (25th, 75th) quantiles for continuous variables. ^a^ Note, not all variables were available from HC, and data represent normal range at the laboratory of Medical Biochemistry, Oslo University Hospital, Oslo, Norway. For P/F ratio there are no “normal range”, but we have previously defined P/F ratio below 26.6 kPa as severe respiratory failure in this COVID-19 cohort (20).

### 2.2 Ethical considerations

The study was approved by the Committee for Medical Research Ethics Region Southeast Norway (approval no. 118684) and the Norwegian Medicines Agency (20/04950–23) and was performed according to the Declaration of Helsinki. All participants gave informed consent before inclusion, directly or through a legally authorised representative.

### 2.3 Isolation of peripheral blood mononuclear cells

Whole blood was collected in BD CPT™ Cell Preparation Tube (Becton, Dickinson, Franklin Lakes, NJ) coated with sodium heparin, and PBMC was isolated following the manufacturer’s instruction. Briefly, CPT tubes were mixed by inverting the tube eight times and centrifuged (1650 G, 15 minutes, RT). The PBMC layer was transferred to a new tube (Corning^®^ 50 mL centrifuge tubes, Merck) and washed twice with PBS, followed by centrifugation (300 G, 15 minutes, RT). After isolation, PBMC pellets without any storage solution were snap-frozen and stored at −80°C until RNA extraction for RNA sequencing analyses. For a subset of the samples (i.e., 5 mill PBMC), the cells were used for flow cytometry analysis immediately after the cells were collected.

### 2.4 Immune cell characterisation by flow-cytometry

Routine blood samples were taken with tubes coated with sodium heparin during hospitalisation and used for leukocyte differential count measurements on Sysmex XN-10 (Sysmex, Kobe, Japan). Additionally, PBMC aliquoted from CPT tubes was used for 8-colour Immunophenotyping by an anti-human REAfinity™ kit (Miltenyi Biotec) for subsequent flow cytometry analyses using the MACSQuant^®^ Analyzer 10 (Milteny Biotec).

### 2.5 Enzyme-linked immunosorbent assay

Plasma levels of B-cell-activating factor (BAFF), soluble (s)CD25, and soluble T cell immunoglobulin and mucin domain-containing protein 3 (sTIM-3) were measured in duplicate by enzyme immunoassays using commercially available antibodies in a 384-format using a combination of a SELMA pipetting robot (Analytik Jena AG, Jena, Germany) and a BioTek dispenser/washer (BioTek Instruments, Winooski, VT). Absorption was read at 450 nm using an enzyme immunoassay plate reader (BioTek Instruments) with wavelength correction set to 540 nm.

### 2.6 Meso scale discovery

The protein level in plasma was quantified for interleukin (IL) IL-6, IL-8, IL-22, IL-23, and interferon-inducing protein (IP)-10/CXCL10 using a U-plex Immuno-oncology Group 1 (Human) assay kit from Mesoscale Discovery (Rockville, MD, USA) utilising a QuickPlex SQ120 according to the manufacturer’s instructions and analysed on a MESO QuickPlex SQ 120MM reader.

### 2.7 RNA sequencing of PBMC

Total RNA was isolated from PBMC of 15 COVID-19 patients (see **Table 1** for RNA-seq cohort demographics) and six controls using the miRNeasy Kit (Qiagen, Hilden, Germany), following the manufacturer’s instructions. Per the manufacturer’s recommendation, isopropanol was added to the RW1 wash buffer to increase yield. RNA was separated from the DNA and protein fraction by phase separation. The aqueous phase containing RNA was loaded on RNA columns for column-based RNA isolation and eluted in nuclease-free water. After isolation, RNA concentration and purity were determined by spectrophotometer absorbance (NanoDrop ND-1000, Thermo Fisher Scientific) based on their 260/230 and 260/280 ratios. The RNA was then stored at −80°C until further analysis.

Total RNA was sent to Novogene (UK) Company Limited for stranded bulk library preparation and RNA sequencing (RNA-seq) on the Illumina platform. The fastp software (v0.20.1) (21) removed contaminated adapters and low-quality reads with a phred score below 30 in the pair-end 150 bp raw sequencing files. Mapping of filtered reads to the Gencode transcriptome H38 (https://www.gencodegenes.org/human/release_38.html) was performed with Salmon (v1.5.2) (22) with 200 bootstrap iterations. Genes were refined to include only protein-coding genes and genes with more than ten counts across all samples. To obtain the differentially expressed genes (DEGs), the Salmon outputs were imported into DESeq2 (v1.38.3) (23) via tximport (v.1.26.1) (24).

### 2.8 Statistical analyses

#### 2.8.1 Analyses of RNA sequencing of PBMC

All statistical analyses were performed in Rstudio (v.2022.12.0 + 353) with R version 4.2.1. Through tximeta (v1.16.1) (25), Salmon outputs were summarised to the gene level for differential gene expression analysis and gene set enrichment analysis (GSEA). Normalisation of gene levels was performed in DESeq2; however, before formal analysis, we applied a batch correction method with the *sva* package to detect underlying unexplained variance seen as surrogate variables (SVs) (26). This adjustment was performed on two separate occasions. Initially, we compared COVID-19 samples to healthy controls, and within that dataset, out of 5 detected SVs, we included the first three to our data before proceeding with formal analysis (**Supplementary Figure S1**; **Supplementary Table S2**). Following our second comparison, where we compared ICU-treated patients to ward patients, we included all 3 identified SVs in the dataset to adjust for unknown batch effects (**Supplementary Figure S2**; **Supplementary Table S3**).

In the formal statistical analysis of PBMC DEGs, we employed DESeq2, a widely utilised tool for RNA-seq data, which models gene expression counts using a negative binomial distribution. DEGs were derived from DESeq2 with the contrast function between the groups of interest, where genes with a p-value <0.05 were considered DEGs.

For GSEA, we applied the *fgsea* (v.1.24.0) (27) package on normalised counts, pre-ranked by their log2fold change. GSEA parameters were set to a cut-off value of min=15 and max=500 genes to assess enrichment of the gene sets “NABA ECM REGULATORS”, “NABA ECM AFFILIATED”, “BIOCARTA ECM PATHWAY”, and “REACTOME NON INTERGRIN MEMBRANE ECM INTERACTION”. Gene sets were derived from the Molecular signature database (MSigDB, version 7.5.1). Pearson correlation analysis of ECM remodelling gene expression and clinical- and circulating variables was performed in R, with the cor() function and presented with the *corrplot* package (v.0.92) (28).

#### 2.8.2 Analyses of publicly available RNA sequencing data from lung tissue of COVID-19 patients

Raw gene expression counts of post-mortem pulmonary tissue (GSE164013) samples derived from deceased COVID-19 patients were downloaded from the public repository Gene Expression Omnibus (GEO) (https://www.ncbi.nlm.nih.gov/geo/). Normalisation and gene expression analysis were performed with DESeq2 without the addition of surrogate variable analysis and batch correction. Normalised genes, pre-ranked by their log2fold change, were then imported into the *fgsea* package for gene set enrichment analysis.

#### 2.8.3 Analyses of publicly available proteome data of PBMC in COVID-19 patients

Pre-processed PBMC proteomics data from COVID-19 patients (n=17), collected on day 7 of hospitalisation, was obtained through a recent publications GitHub repository (https://github.com/GiuseppeLeite/COVID19_Proteomic) (29). Before differential protein abundance analysis, data were normalised, log2FC transformed, filtered, imputed and batch effect corrected as described in the original article (29). Sample clustering was inspected with a multidimensional scaling (MDS) plot (**Supplementary Figure S3**). Differential protein abundance analysis was performed using the R/Bioconductor package *Limma* (version 3.54.2) (30, 31), and GSEA was performed using the *fgsea* package on log2fold change pre-ranked proteins. Proteins with a p-value <0.05 were deemed significantly abundant.

In addition to R, Inkscape 0.92.4 was used for final adjustments of the result figures involved in ECM remodelling and regulation.

## 3 Results

### 3.1 Transcriptome analysis of PBMC reveals heightened expression of ECM remodelling genes in individuals with COVID-19

**Figure 1:**
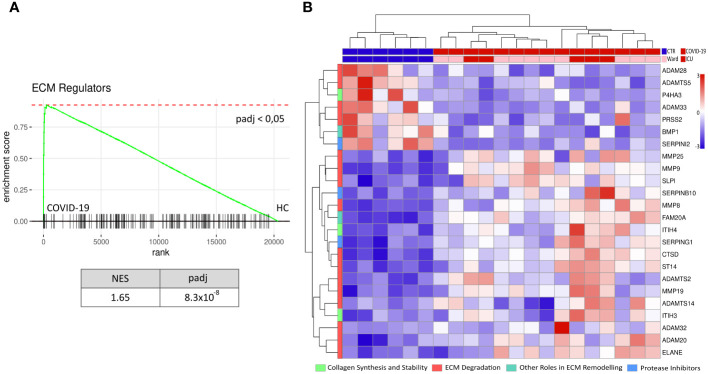
Increased ECM remodelling in PBMC during COVID-19. RNAseq data of peripheral mononuclear cells (PBMC) from hospitalised COVID-19 patients (n=15) and age-matched healthy controls (n=6) was tested against the gene set NABA ECM REGULATORS for enrichment **(A)**. PBMC from COVID-19 patients showed an increased enrichment of ECM regulators compared to the healthy controls (HC). Further, the normalised level of the 24 genes in this pathway with a significant differential gene expression (p-value< 0.05, log2fold > 1 & log2fold < -1), categorised by their biological function (i.e., collagen synthesis and stability, ECM degradation, other roles in ECM remodelling and protease inhibitors), is presented as a heat map **(B)**. NES = normalised enrichment score, padj = p-value corrected for multiple tests, rank = log2fold change ranked genes (COVID-19 vs HC).

We first performed RNA-seq in PBMC isolated from hospitalised COVID-19 patients (n=15) and age-matched healthy controls (n=6) with a focus on pathways related to ECM remodelling (**Supplementary Figure S4**). Of the ECM-related gene sets analysed, we observed an increase in the enrichment of genes associated with ECM regulatory function (NABA ECM REGULATORS) compared to healthy controls (**Figure 1A**). Of 238 genes allocated to this gene set, we detected 169 in our data (**Supplementary Figure S5**). We further investigated the expression pattern of the most regulated genes belonging to the NABA ECM REGULATORS gene set, categorised by their role in ECM remodelling. In general, most of the DEGs were upregulated in COVID-19 patients compared to healthy controls (**Figure 1B**). No clear regulation pattern of the selected ECM remodelling categories (i.e., “ECM Degradation”, “Collagen Synthesis, “Other roles in ECM remodelling”, and “Protease Inhibitors”) was observed. However, most DEGs belonged to the “ECM Degradation” category.

### 3.2 ECM remodelling genes modified with disease severity in hospitalised COVID-19 patients

**Figure 2:**
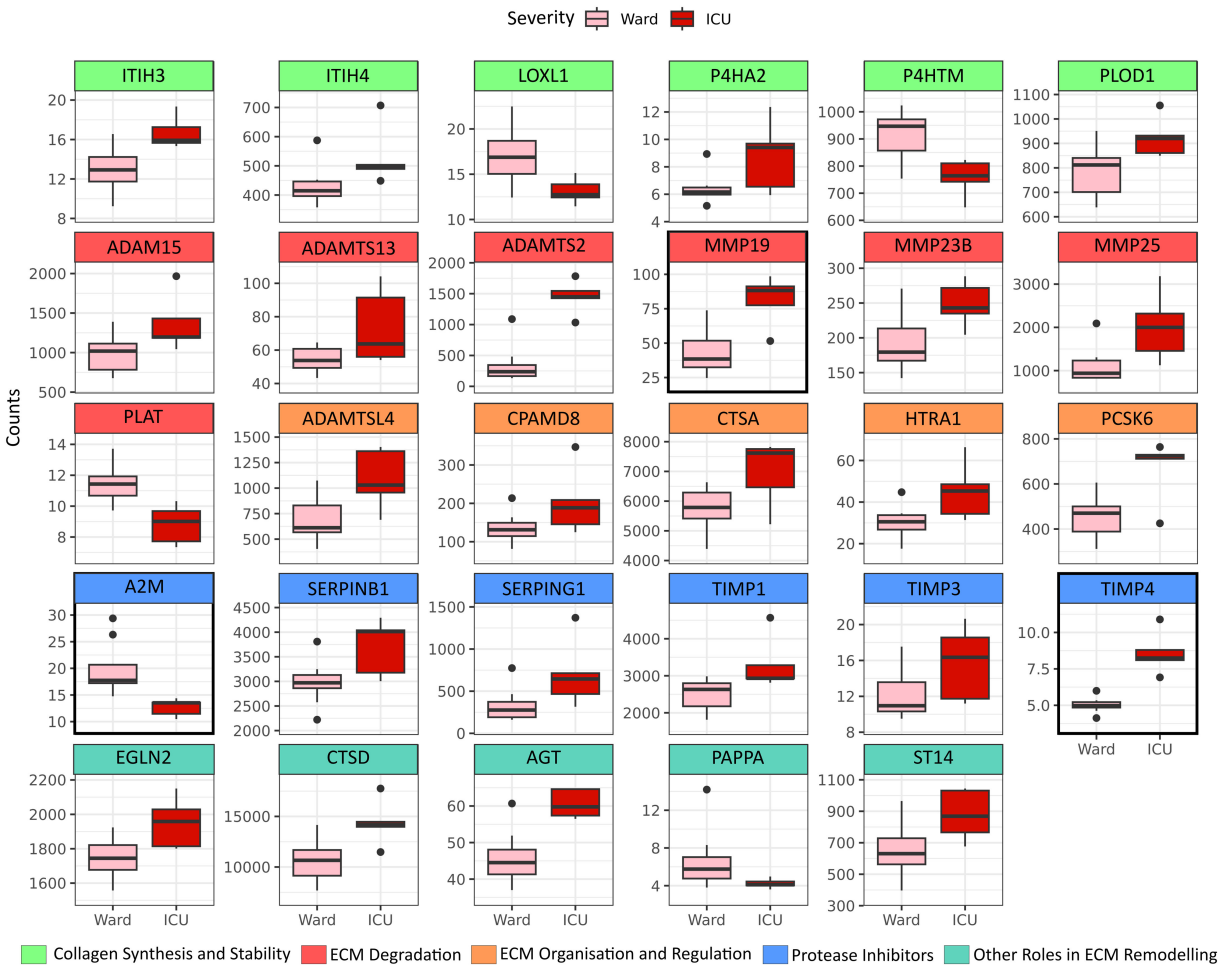
ECM modulators increased in PBMC by COVID-19 severity. Differentially expressed genes belonging to the ECM-regulator gene set (NABA ECM REGULATORS), defined by p-value <0.05 when comparing peripheral blood mononuclear cells (PBMC) isolated from patients in need of Intensive Care Unit (ICU) treatment (N=5) to PBMC isolated from WARD patients (N=10). Genes are grouped by their functional role in ECM remodelling. Genes with a marked black square are significantly regulated also when considering multiple testing against the whole transcriptome (padj <0.05).

When comparing PBMCs of patients requiring ICU treatment (n=5) with those treated at the hospital ward (n=10), we identified 29 differentially expressed transcripts within the ECM-regulator gene set (NABA ECM REGULATORS, **Figure 2**), subsequently referred to as severity-associated genes. A summary of the individual genes and their abbreviations is presented in **Supplementary Table S4**. Genes were grouped according to their assumed role in ECM remodelling. Genes belonging to the group; “Collagen synthesis and Stability” were overall increased in ICU patients (ITIH3, ITIH4, P4HA2, PLOD1), except LOXL1 and P4HTM which were less expressed in ICU patients. Genes related to “ECM Degradation” containing several MMPs (MMP-19, MMP-23B, MMP-25) and members of the adamlysin family (ADAM-15, ADAMTS-13, ADAMT-S2) were, in general, up-regulated in the ICU as compared to ward patients, except for the Plasminogen Activator Tissue Type (PLAT) which was downregulated in ICU patients. ICU patients showed upregulation of all genes in the “ECM Organisation and Regulation” group (i.e., ADAMTSL4, CPAMD8, CTSA, HTRA1, PCSK6). In the “Protease Inhibitors” group, we observed that, except for the Alpha-2-Macroglobulin (A2M) gene, all genes were significantly upregulated in ICU patients (SERPINB1, SERPING1, TIMP-1, TIMP-3, TIMP-4). Finally, the last group, termed “Other Roles in ECM Remodeling”, was again upregulated in ICU patients compared to those treated at the hospital ward (EGLN2, CTSD, AGT, ST14) apart from pappalysin (PAPPA), which was less expressed in ICU patients. When accounting for multiple testing in consideration of the whole transcriptome, only MMP-19, A2M, and TIMP-4 remained significant (padj < 0.05).

### 3.3 Association of clinical characteristics, monocyte and lymphocyte subpopulation and viral load with severity-driven ECM remodelling genes

**Figure 3:**
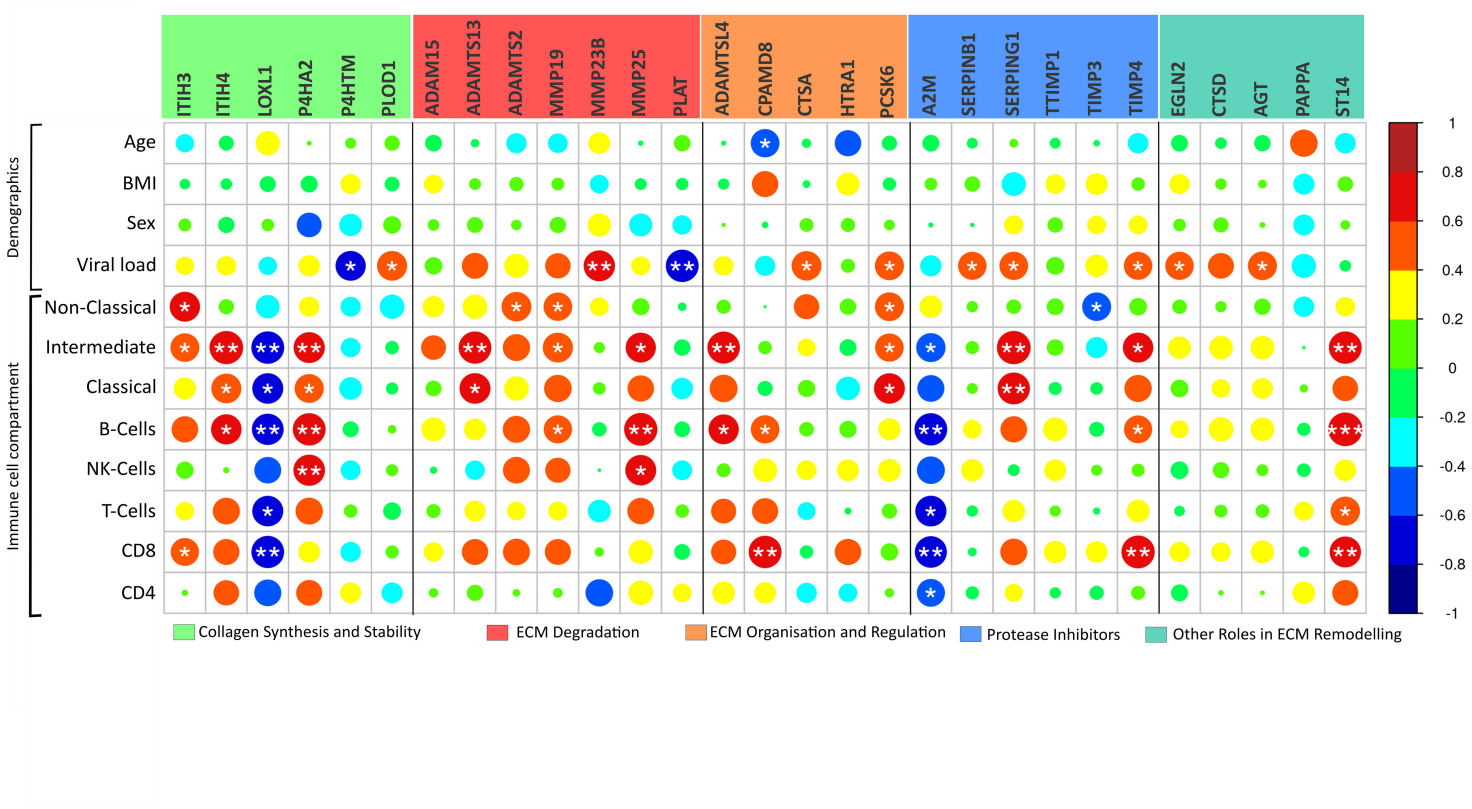
ECM modulators in relation to clinical parameters. Correlation analysis of the 29 COVID-19 severity-associated ECM genes in peripheral blood mononuclear cells (PBMC) and clinical parameters reveal a relationship between immune cell compartments and viral load. Notably, “Collagen synthesis and Stability” grouped genes present a particular connection to immune cell compartments. Genes are grouped by their functional role in ECM matrix remodelling. Body mass index (BMI); Viral load, viral copies/µL in oropharyngeal sample. Immune cell compartment; Non-Classical, non-classical monocytes; Intermediate, intermediate monocytes; Classical, classical monocytes. *p < 0.05, **p < 0.01, ***p < 0.001.

While age, BMI, and sex displayed minimal correlations with the identified severity-driven ECM remodelling genes, viral load exhibited significant associations with 11 identified ECM remodelling mediators, where nine were positively correlated (**Figure 3**).

PBMC represents subsets of lymphocytes and monocytes. For several of the immune cell subsets, there was a positive association with gene expression of the identified ECM remodelling genes (**Figure 3**). Yet, LOXL1 and A2M expression was generally negatively associated with cell subsets, and in general, few associations were seen concerning the number of CD4^+^ T cells and NK cells (**Figure 3**).

### 3.4 Associations of circulating inflammation markers with ECM remodelling genes according to disease severity

**Figure 4:**
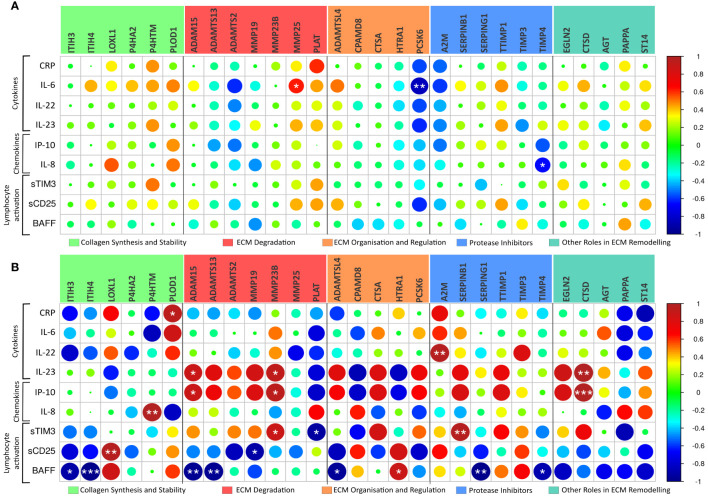
ECM modulators related to circulating cytokines. Pearson correlation analysis of the 29 COVID-19 severity-associated ECM genes in peripheral blood mononuclear cells (PBMC) and circulating cytokines/chemokines, as assessed by ELISA and MSD, reveals a distinct relationship between the immune system and ECM regulator genes in Ward patients (**(A)**, n=10) and ICU patients (**(B)**, n=5). CRP, C-reactive protein; sTIM-3, soluble cell immunoglobulin and mucin domain-containing protein 3; BAFF, of B-cell-activating factor. *p < 0.05, **p < 0.01, ***p < 0.001.

We next investigated the association between plasma levels of markers of inflammation and immune activation, as assessed by ELISA and MSD, and the gene expression of severity-associated genes involved in ECM remodelling in PBMCs from the same patients (see **Figure 4**). In general, there was a much stronger correlation between the expression levels of ECM regulators and markers of inflammation and immune activation, in patients treated at the ICU (n=5) than in patients treated in the hospital ward (n=10). Several significant associations were revealed in the ICU patients, and of particular interest, BAFF as a marker of B cell activation was negatively associated with multiple regulators of ECM remodelling (**Figure 4**). Notably, the number of B cells was positively associated with several ECM markers (**Figure 3**). In contrast to BAFF, inflammatory cytokines (i.e., IL-22 and, in particular, IL-23) and chemokine IL-8/CXCL8 and, to some degree, also sTIM-3 as a marker of T cell exhaustion were positively associated with certain ECM regulators, varying across functional groups.

### 3.5 PBMC of severe COVID-19 patients is enriched in ECM regulator proteins

**Figure 5:**
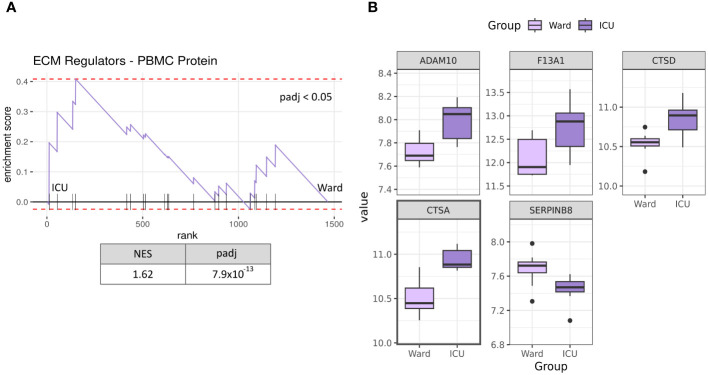
Protein expression of PBMC in severe COVID-19 patients. GSEA analysis of PBMC protein levels from publicly available proteome analyses [28] shows that ECM regulators are significantly enriched in ICU patients (n=9) compared to Ward patients (n=8) **(A)**. Significantly differentially abundant ECM regulator proteins in severe COVID-19 patients (p-value < 0.05, ICU vs Ward) **(B)**. Only CTSA was significant when accounting for multiple tests (padj < 0.05). NES, normalised enrichment score; ICU, intensive care unit: Ward, hospital ward; padj, p-value corrected for multiple testing. Proteins with a marked black square are also significantly regulated when considering multiple tests against the whole proteome (padj <0.05).

Recently, Figueirêdo Leite *et al*. published their proteomics data from PBMC in hospitalised COVID-19 subjects, including both patients treated at the ICU (n=9) and those treated at the hospital ward (n=8) (29). To assess whether the transcriptional changes in PBMC from the present study were representative of changes at the protein level, we next accessed this proteomics dataset to investigate changes in the ECM remodelling process. Our GSEA revealed that the ECM regulators gene set was enriched in ICU patients compared to Ward (**Figure 5**), with a normalised enrichment score of 1.62. A total of 20 ECM regulator proteins were detected in this dataset (**Supplementary Table S5**). Of them, five proteins were significantly differentially abundant between ICU and Ward patients (ADAM10, F1A1, CTSD, CTSA, SERPINB8). Of the regulated proteins CTSD and CTSA genes were also regulated at the mRNA level in ICU patients in our study (see **Figure 2**). When accounting for multiple tests while considering the entire proteomics dataset, only CTSA remained significant (padj <0.05).

### 3.6 Inflamed tissue shows increased ECM remodelling in lungs infected with SARS-CoV-2

**Figure 6:**
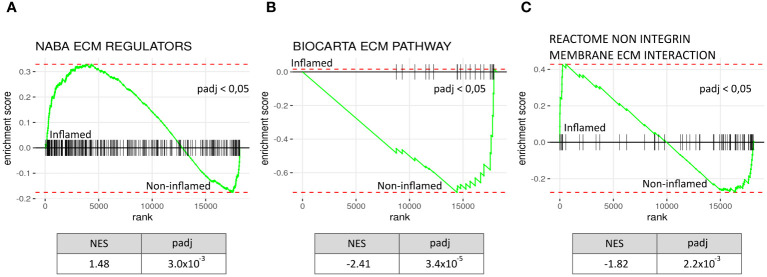
Increased ECM remodelling in inflamed lung tissue from deceased COVID-19 patients. Comparing a publicly available gene expression data set of inflamed lung tissue to paired non-inflamed tissue from the same diseased COVID-19 patients (N=5, inflamed biopsies = 49, non-inflamed biopsies=17) shows ECM regulatory activity when using GSEA analysis on the gene dataset: NABA ECM REGULATORS **(A)**, BIOCARTA ECM PATHWAY **(B)**, and REACTOME NON INTEGRIN MEMBRANE ECM INTERACTION **(C)**. ECM regulators were significantly increased in inflamed tissue with a NES of 1.48; meanwhile Biocarta ECM pathway genes and genes in the Reactome non integrin membrane ECM interaction geneset were decreased. NES, normalised enrichment score, padj = p-value corrected for multiple tests, rank = log2fold ranked genes (inflamed vs non-inflamed).

Finally, we used a publicly available RNA-seq dataset (GSE164013) from five deceased COVID-19 patients, assessing gene set enrichment in 49 biopsies from inflamed lung tissue compared to 17 biopsies from non-inflamed lung tissue (32). GSEA results from all four ECM-related gene sets are presented in **Supplementary Figure S6**. Inflamed tissue exhibited NABA ECM REGULATORS pathway enrichment, similar to PBMCs from hospitalised COVID-19 patients. Other ECM-related signalling pathways, such as BIOCARTA ECM PATHWAY and REACTOME NON-INTEGRIN MEMBRANE ECM INTERACTION, were significantly decreased in inflamed tissue. These findings indicate an interaction between inflammation and ECM remodelling within lung tissue in severely ill COVID-19 patients (**Figure 6**).

## 4 Discussion

We and others have previously reported up-regulation of several markers of ECM remodelling in plasma or serum in relation to disease severity in hospitalised COVID-19 patients (14, 33, 34). In the present explorative study, we extend these findings by showing a marked upregulation of several pathways related to ECM remodelling in transcriptome analyses of circulating immune cells from hospitalised COVID-19 patients, particularly those with the most severe disease. A similar pattern was also seen at the protein levels in PBMC and within the pulmonary tissue of deceased COVID-19 patients when comparing inflammatory and non-inflammatory lung tissue by using publicly available proteome and RNA-seq dataset, respectively. Our findings support that immune cell-driven regulation of ECM is involved in the pathogenesis of severe COVID-19, representing potential novel targets for therapy in this disorder.

Several studies have shown a marked dysregulation of circulating mediators, including MMPs and TIMPs, concerning ECM remodelling in COVID-19 patients (6). We recently proposed S100A12, YKL-40 and osteopontin as circulating markers of ECM remodelling. Increased levels of these markers during hospitalisation were associated with more severe disease and pulmonary pathology at 3-month follow-up as assessed by low-dose chest computed tomography (CT) images (14). Herein, the transcriptome analyses of PBMC show the potential pathogenic involvement of a range of other mediators of ECM remodelling in hospitalised COVID-19 patients. Several members of the ADAM and ADAMTS families, known to be involved in various pulmonary disorders (35, 36), were up-regulated in COVID-19 patients who needed treatment at the ICU compared with those treated at the hospital ward. Moreover, in COVID-19, much focus has been directed against MMP-9 (13, 37–39). However, the human MMP family consists of several genes, and herein we show increased transcript levels of MMP-19, MMP-23B and MMP-25 in relation to disease severity in PBMC from COVID-19 patients, underscoring a role for MMPs beyond that of MMP-9 in COVID-19. In particular MMP-19 and TIMP-4 were found to be dysregulated also after full adjustment for multiple comparison. Whereas data on MMP-19 in COVID-19 are scarce, it has been related to enhanced fibrogenesis in pulmonary tissue (40). As for TIMP-4, the potential implications of enhanced expression in PBMC are at present unclear, but as TIMPs are known to inhibit certain MMPs, it could potentially represent a counteracting mechanism to attenuate the MMP-mediated effects on ECM degradation as suggested in chronic obstructive pulmonary diseases (41). However, TIMP-4 seems to have properties beyond and independent of MMP inhibition such as effects on cell proliferation and apoptosis that clearly could be relevant in relation to COVID-19 (42). Nonetheless, our preliminary findings may suggest that a multitude of mediators involved in ECM remodelling are regulated in severe COVID-19, reflecting a complex network interacting with the immune cells and their inflammatory mediators.

In addition to MMP-19 and TIMP-4, A2M was also associated with severe disease even after adjustment for multiple testing. Although A2M is an established broad spectrum protease inhibitor with well-known role in inflammation, immunity and infections, including pulmonary disorders, its mechanisms of action is still not clear (43). In the present study we found an association between A2M and plasma levels of inflammatory cytokines. Notably, A2M may act as a reservoir for cytokines and growth factors, including IL-6, which we found to be associated with A2M. Potentially increasing the half-life of these cytokines (43, 44). However, further studies on the role of A2M in COVID-19, is needed to clarify this. Additionally, A2M has also been suggested to counteract thromboinflammation in COVID-19 (45) and further studies are clearly needed to elucidate the role of A2M in COVID-19.

SARS-CoV-2 could potentially, through interaction with its receptor, angiotensin-converting receptor 2, modulate the expression of mediators of ECM remodelling (9). Indeed, we have recently shown that inactivated SARS-CoV-2 enhanced the release of Galectin 3 and MMP-9 in macrophages and growth differentiating factor 15 in lung epithelial and alveolar type 2 cells (14). In the present study, we find that the viral load of SARS-CoV-2 in the oropharynx was correlated with transcript levels of several ECM mediators. This could suggest that SARS-CoV-2 contributes to the marked dysregulation of mediators of ECM remodelling in PBMC, as seen in the present study. However, it is not likely that high viral activity is the predominant driver of ECM regulation. It is more likely that viral-triggered immune dysregulation could play an essential role in these processes.

Not surprisingly, we found a significant association between several inflammatory cytokines/chemokines (e.g., IP-10, IL-22 and IL-23) and markers of T cell activation (sTIM-3) and up-regulation of transcript for several mediators of ECM remodelling. However, BAFF as a marker and mediator of B cell activation (46, 47) was negatively associated with several of these. Indeed, B cells and BAFF have been shown to stimulate collagen synthesis and possess pro-fibrotic properties (48). B cells also seem to have protective activities during acute exacerbations of chronic obstructive pulmonary disease (49). However, the role of BAFF in ECM remodelling in severe COVID-19 disease still needs to be clarified.

ECM degradation is not necessarily harmful. It is demonstrated that T cells tend to navigate more readily through tissue areas with a less dense ECM structure (50). Further, studies have revealed that enzymatic degradation of glycocalyx components within the ECM can uncover endothelial adhesion molecules. This, in turn, promotes heightened interactions between leukocytes and endothelial cells (51–53). Nonetheless, if the ECM experiences damage or alteration prompted by trauma or degradation, it can trigger the liberation of ECM fragments and cytokines bound within the ECM structure (54). These substances can potentially trigger a local inflammatory immune response, leading to the recruitment of chemotactic immune cells and subsequent inflammation (55). Despite this knowledge, the intricate interaction between ECM, ECM fragments, and leukocytes remains a topic yet to be wholly understood (9, 56), including the role of these complex interactions in COVID-19.

The present explorative study has important limitations, such as a relatively low sample size, particularly patients in the ICU and healthy controls. Although we have included analyses of datasets from other studies on pulmonary tissue (RNA-seq) and PBMC (proteome), confirming the upregulation of the actual pathways related to ECM remodelling in different patient cohorts, we did not confirm up-regulation of the most central molecules like MMP-19, TIMP-4 and A2M. Moreover, data based on peripheral material, such as in the present study, are never fully representative of *in situ* processes; however, due to the ethical issues arising from sampling pulmonary tissue in critically ill patients, we opted to use peripheral samples as a proxy. Furthermore, the pre-defined aim of this study was to investigate the regulation of mediators of ECM remodelling in PBMC of hospitalised COVID-19 patients in relation to disease severity using an explorative approach. We, therefore first presented data without full adjustment for multiple testing and this may be considered as a limitation of the study. Also, correlations do not necessarily mean any causal relationship. Additionally, several comparisons were performed and hence need further validation.

Nonetheless, our findings emphasise that various factors involved in ECM remodelling may play a role in severe COVID-19. They engage with inflammatory markers and immune cells, impacting the systemic environment and the lungs. The intricate interplay between ECM regulation and inflammation/immune response regulation warrants further investigation to identify novel therapeutic targets for mitigating severe COVID-19 and potentially ameliorating pulmonary damage. Such studies should include a larger number of patients and controls and preferentially also mechanistic studies to uncover the potential role of ECM remodelling in severe COVID-19.

## Data availability statement

The datasets presented in this article are not readily available because of ethical and privacy restrictions. For access to datasets, an institutional data transfer agreement can be established. Data can thus be shared if the aims of data use are covered by ethical approval and patient consent. Requests to access the datasets should be directed to tuvad@medisin.uio.no.

## Ethics statement

The studies involving humans were approved by Committee for Medical Research Ethics Region Southeast Norway (approval no. 118684) and the Norwegian Medicines Agency (20/04950-23). The studies were conducted in accordance with the local legislation and institutional requirements. Written informed consent for participation in this study was provided by the participants’ legal guardians/next of kin.

## Author contributions

SLM: Conceptualization, Formal Analysis, Investigation, Visualization, Writing – original draft, Writing – review & editing, Data curation. NRB: Formal Analysis, Investigation, Methodology, Writing – original draft, Writing – review & editing. TR: Data curation, Formal Analysis, Writing – original draft, Writing – review & editing. ELS: Data curation, Formal Analysis, Investigation, Writing – original draft, Writing – review & editing. CH: Data curation, Writing – original draft, Writing – review & editing. VB: Data curation, Writing – original draft, Writing – review & editing. AEM: Data curation, Formal Analysis, Investigation, Writing – original draft, Writing – review & editing. AKF: Data curation, Project administration, Validation, Writing – original draft, Writing – review & editing. LH: Data curation, Project administration, Writing – original draft, Writing – review & editing. AMDR: Data curation, Project administration, Writing – original draft, Writing – review & editing. AT: Data curation, Project administration, Writing – original draft, Writing – review & editing. ARH: Data curation, Project administration, Writing – original draft, Writing – review & editing. MT: Project administration, Writing – original draft, Writing – review & editing, Data curation. TUe: Conceptualization, Data curation, Formal Analysis, Investigation, Project administration, Writing – original draft, Writing – review & editing. TUl: Formal Analysis, Methodology, Writing – original draft, Writing – review & editing. PA: Conceptualization, Funding acquisition, Project administration, Writing – original draft, Writing – review & editing. ABD: Conceptualization, Data curation, Funding acquisition, Project administration, Writing – original draft, Writing – review & editing. BH: Conceptualization, Data curation, Funding acquisition, Project administration, Resources, Writing – original draft, Writing – review & editing. TBD: Conceptualization, Data curation, Investigation, Project administration, Supervision, Writing – original draft, Writing – review & editing.
