# Supplementary material for: Extracellular matrix remodelling pathway in peripheral blood mononuclear cells from severe COVID-19 patients: an explorative study
Source: Front Immunol. 2024 Jun 18;15:1379570. doi: 10.3389/fimmu.2024.1379570 (PMC11217192; doi:10.3389/fimmu.2024.1379570)
Supplement: Supplementary file 1 [file DataSheet_1.docx]

**Supplemental Table S1.** Demographic, clinical, and biochemical characteristics in 147 patients hospitalised for COVID-19 included in the original NOR-Solidarity cohort. Further, 15 patients from the primary cohort were included in the present study's RNA-seq analysis (RNAseq Cohort).

| **Parameter** | **Main Cohort**  **n=147** | **RNAseq Cohort**  **n=15** |
| --- | --- | --- |
| Age, years | 59.6±15.5 | 60.0±13.4 |
| Male gender (%) | 91 (62) | 12 (80) |
| Body Mass Index, kg/m2 | 28.1±4.6 | 29.5±4.7 |
| Vaccinated ≥ 1 dose (%) | 0 (0) | 0 (0) |
| Dexamethasone (%) | 1 (1) | 1 (7) |
| Comorbidities |  |  |
| Chronic cardiac disease (%) | 24 (16) | 0 (0) |
| Hypertension (%) | 46 (32) | 5 (36) |
| Chronic pulmonary disease (%) | 28 (19) | 4 (29) |
| Obesity (%) | 37 (28) | 6 (40) |
| Diabetes (%) | 24 (16) | 3 (21) |
| Current smoker (%) | 3 (2) | 2 (14) |
| HIV (%) | 8 (5) | 0 (0) |
| Oxygen therapy (%) | 81 (55) | 11 (73) |
| Outcomes |  |  |
| ICU admission (%) | 26 (18) | 5 (33) |
| P/F-ratio at admission, kPa | 42.4 (32.9,49.6) | 39.6 (30.4, 45.4) |
| Haemoglobin, g/dL | 13.1±1.6 | 13.6± 1.1 |
| C-reactive protein, mg/L | 69 (34,131) | 85 (48, 161) |
| Ferritin, µg/L | 619 (368,1115) | 586 (293, 1065) |
| White blood cell count, x 10^9^/L | 6.4±2.6 | 7.8±4.0 |
| Neutrophils, x 10^9^/L | 4.7±2.5 | 6.3±4.1* |
| Lymphocytes, x 10^9^/L | 1.2±0.54 | 1.0±0.4 |
| eGFR | 87±24 | 93.3±25.9 |

ICU; intensive care unit, P/F-ratio; PaO2/FiO2-ratio, eGFR; estimated glomerular filtration rate. Values are presented as mean ± SD or median (25^th^, 75^th^) quantiles for continuous variables. * p-value <0.05, t-test.

**Supplemental Figure S1. Batch correction of COVID-19 and healthy control samples.**


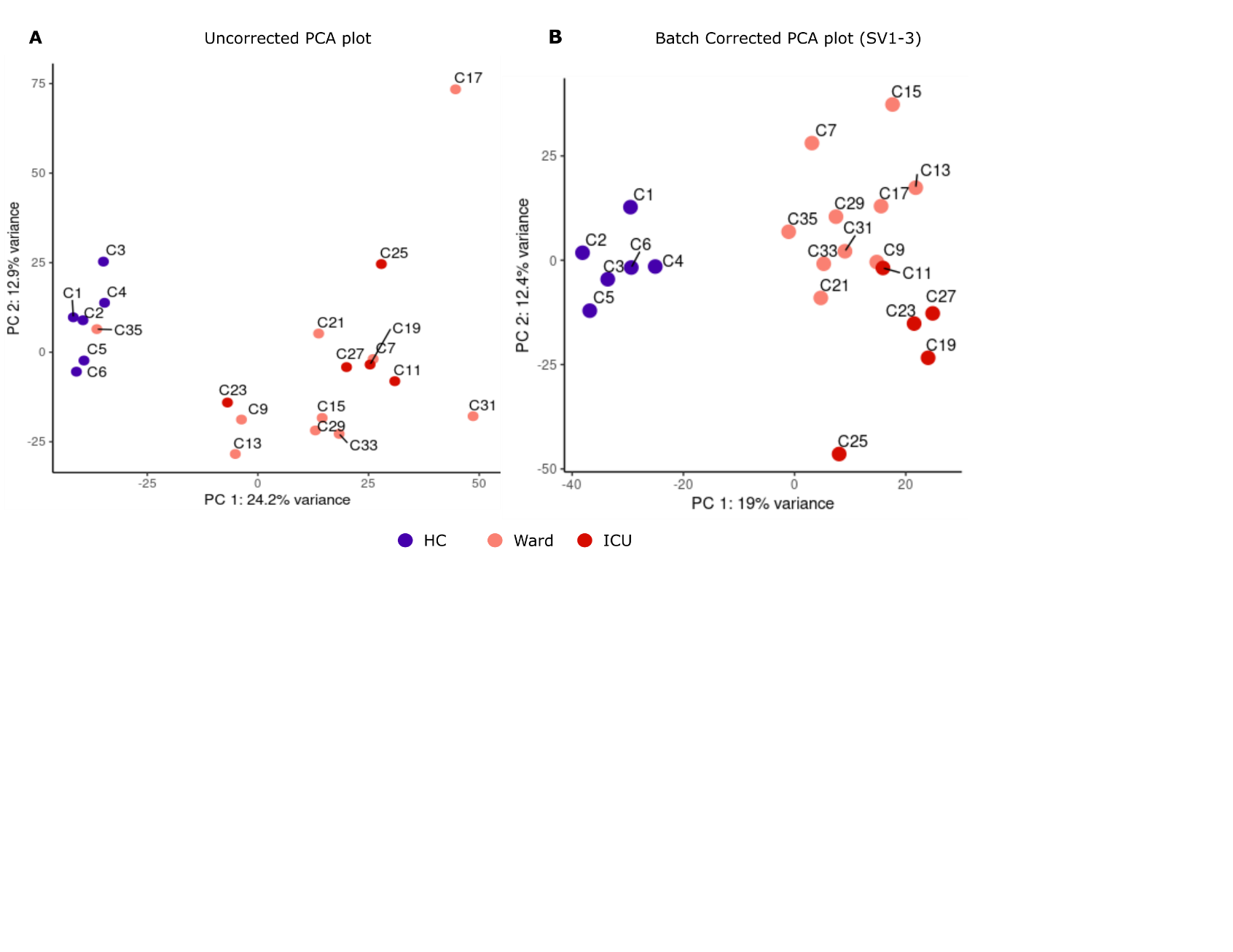


Principal component analysis (PCA) of variance stabilised, uncorrected normalised counts (**A**). ~~Principal component analysis (~~PCA~~)~~ of variance stabilised, batch-corrected with surrogate variables package (SVA), normalised counts. Of 5 surrogate variables (SV) detected, SV1, SV2 and SV3 are included (**B**). HC = healthy control, Ward = hospitalised ward patients, ICU = Intensive Care Unit patients.

**Supplemental Table S2. Sample metadata and surrogate variables of PBMC from COVID-19 patients and healthy controls**

Sample metadata and surrogate variables detected during the batch correction, before formal analysis, of COVID-19 PBMC versus healthy controls comparison.

| ID | Condition | Type | SV1 | SV2 | SV3 | SV4 | SV5 |
| --- | --- | --- | --- | --- | --- | --- | --- |
| C1 | HC | CTR | -0,22773 | 0,155271 | 0,14606 | -0,19177 | 0,225017 |
| C2 | HC | CTR | -0,2079 | 0,13188 | -0,23785 | 0,059099 | -0,01114 |
| C3 | HC | CTR | -0,19406 | 0,28029 | -0,29888 | 0,080281 | 0,189852 |
| C4 | HC | CTR | -0,22237 | 0,18311 | -0,24417 | 0,083828 | 0,020913 |
| C5 | HC | CTR | -0,20404 | -0,00241 | 0,224483 | 0,144015 | -0,15342 |
| C6 | HC | CTR | -0,24722 | 0,009935 | 0,23389 | 0,009722 | -0,16212 |
| C7 | Ward | COVID-19 | 0,22327 | -0,07844 | 0,071062 | -0,33974 | 0,528621 |
| C9 | Ward | COVID-19 | -0,07317 | -0,14199 | 0,186455 | 0,025413 | -0,16677 |
| C11 | ICU | COVID-19 | 0,192626 | -0,15899 | 0,077941 | -0,06883 | 0,083925 |
| C13 | Ward | COVID-19 | -0,11977 | -0,18089 | 0,17584 | -0,20164 | -0,20286 |
| C15 | Ward | COVID-19 | 0,080694 | -0,08388 | -0,04227 | -0,4537 | -0,33178 |
| C17 | Ward | COVID-19 | 0,546265 | 0,670171 | 0,043735 | -0,15181 | -0,26989 |
| C19 | ICU | COVID-19 | 0,138459 | -0,1103 | 0,119826 | 0,125555 | -0,11733 |
| C21 | Ward | COVID-19 | 0,047671 | 0,021655 | -0,35224 | 0,174114 | 0,133009 |
| C23 | ICU | COVID-19 | -0,17548 | -0,04879 | -0,18328 | 0,147309 | -0,3792 |
| C25 | ICU | COVID-19 | 0,23945 | 0,054126 | 0,285526 | 0,658635 | 0,119776 |
| C27 | ICU | COVID-19 | 0,089137 | -0,06242 | 0,124468 | -0,01243 | -0,00095 |
| C29 | Ward | COVID-19 | -0,00098 | -0,22365 | -0,34534 | -0,10175 | -0,03732 |
| C31 | Ward | COVID-19 | 0,302613 | -0,3124 | -0,35831 | 0,086771 | 0,07542 |
| C33 | Ward | COVID-19 | 0,102252 | -0,3072 | 0,16133 | 0,043407 | 0,119009 |
| C35 | Ward | COVID-19 | -0,28973 | 0,204931 | 0,21172 | -0,11649 | 0,337243 |

Type = Sample biotype; CTR = control group; COVID-19 = COVID-19 patients. Condition = sample condition; HC= healthy control; ward= ward patient; ICU = intensive care unit patient.SV= surrogate variable.

**Supplemental Figure S2. Batch correction of ICU and Ward samples.**


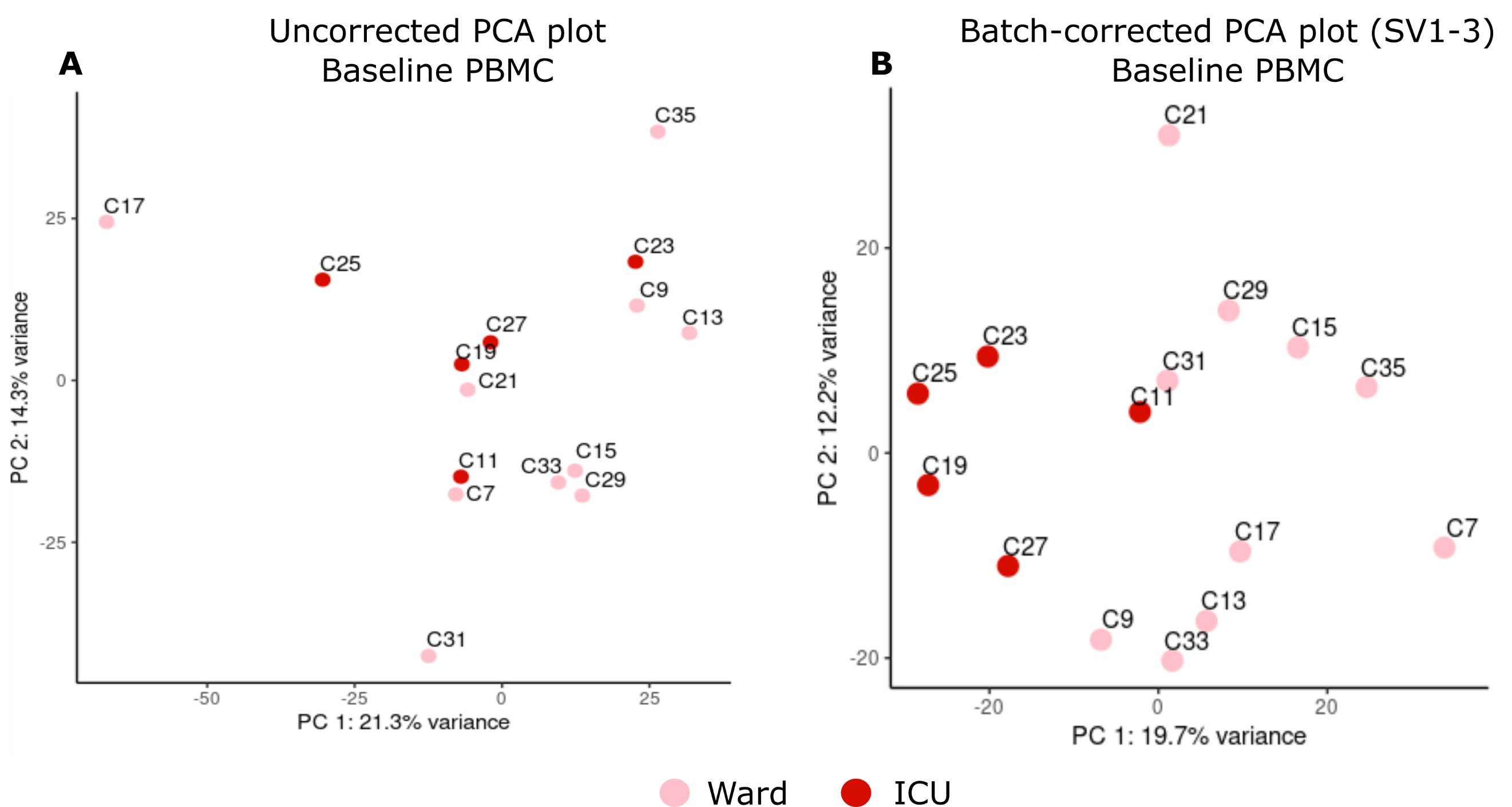


Principal component analysis (PCA) of variance stabilised, uncorrected normalised counts of ICU and WARD samples (**A**). PCA ~~Principal component analysis (PCA)~~ of batch-corrected variance stabilised normalised counts. Of the 3 surrogate variables (SV) detected, SV1, SV2 and SV3 are included to adjust for unknown batch effects (**B**). Ward = hospitalised ward patients, ICU = Intensive Care Unit patients.

**Supplemental Table S3. Sample metadata and surrogate variables of PBMC from ICU- and Ward patients.**

Sample metadata and surrogate variables were detected during the batch correction of ICU and Ward's patient samples, performed before formal analysis.

| ID | Condition | Type | SV1 | SV2 | SV3 |
| --- | --- | --- | --- | --- | --- |
| C19 | ICU | COVID-19 | -0,04063 | 0,008119 | 0,016857 |
| C17 | Ward | COVID-19 | -0,76182 | -0,33309 | -0,34301 |
| C31 | Ward | COVID-19 | -0,12569 | 0,461012 | 0,100303 |
| C9 | Ward | COVID-19 | 0,216903 | -0,11192 | -0,07074 |
| C11 | ICU | COVID-19 | -0,06013 | 0,186968 | 0,065267 |
| C13 | Ward | COVID-19 | 0,308144 | -0,02828 | -0,18842 |
| C15 | Ward | COVID-19 | 0,053602 | 0,153358 | -0,5105 |
| C23 | ICU | COVID-19 | 0,234296 | -0,27442 | -0,21993 |
| C35 | Ward | COVID-19 | 0,308148 | -0,57189 | 0,234803 |
| C29 | Ward | COVID-19 | 0,151829 | 0,220816 | -0,13339 |
| C25 | ICU | COVID-19 | -0,24524 | -0,1864 | 0,586138 |
| C33 | Ward | COVID-19 | 0,103644 | 0,222944 | 0,212565 |
| C27 | ICU | COVID-19 | -0,00578 | 0,003452 | -0,04342 |
| C21 | Ward | COVID-19 | -0,05357 | -0,01312 | 0,070467 |
| C7 | Ward | COVID-19 | -0,08371 | 0,262465 | 0,223013 |

Type = Sample biotype; COVID-19 = COVID-19 patients. Condition = sample condition; ward= ward patient; ICU = intensive care unit patient. SV = surrogate variable.

**Supplemental Figure S3. Severe COVID-19 Proteomics samples.**


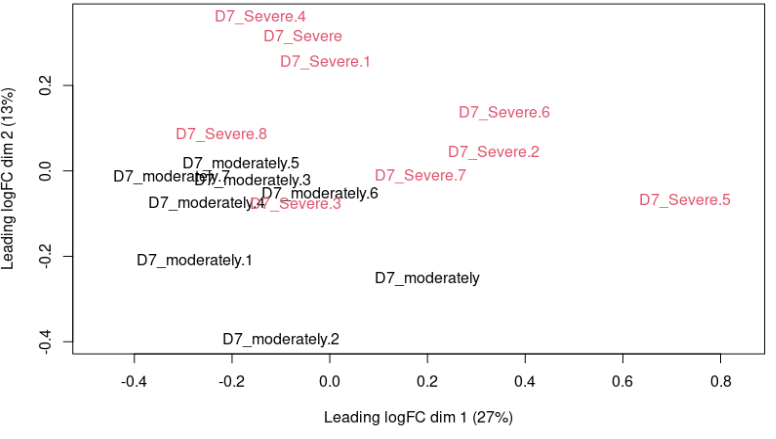


A Multidimensional Scaling (MDS) plot of normalised, log2foldchange transformed, imputed, and batch-corrected proteomics data from PBMC collected during the first week of hospitalisation (D7). Moderate = hospital ward patients, Severe = intensive care unit patients.

**Supplemental Figure S4. Regulation of ECM-related ~~geneset~~ gene sets in PBMC.**


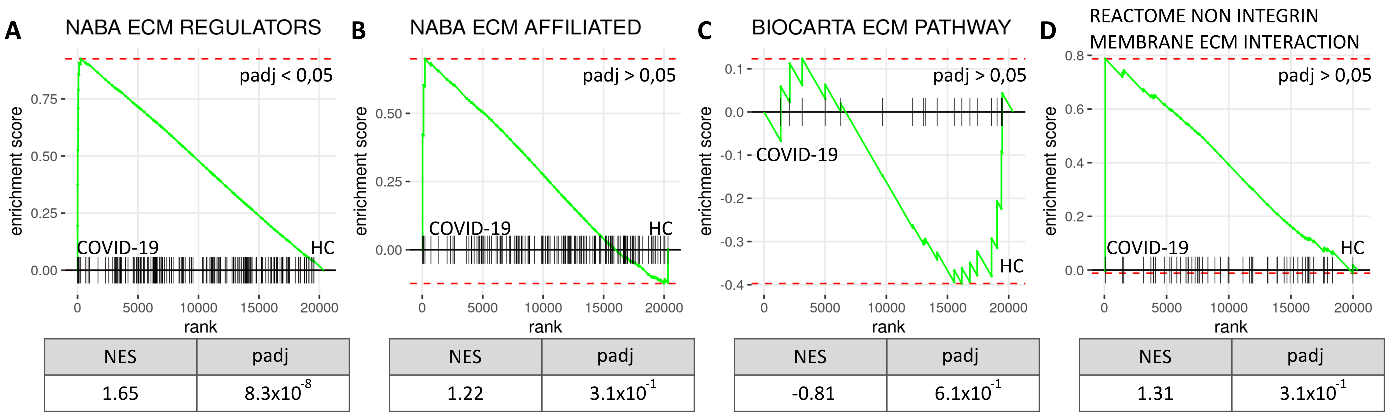


RNAseq data of PBMC from hospitalised COVID-19 patients (n=15) and age-matched healthy controls (n=6) was tested against the gene sets NABA ECM REGULATORS (**A**), NABA ECM AFFILIATED (**B)**, BIOCARTA ECM PATHWAY (**C**) and REACTOME NON INTERGRIN MEMBRANE ECM INTERACTION (**D**) for enrichment. PBMC from COVID-19 patients showed an increased enrichment of ECM regulators compared to the healthy controls (padj < 0.05). padj= p-adjusted value, HC=healthy controls.

**Supplemental Figure S5.** **Gene expression of ECM Regulator genes in COVID-19 PBMC.**

169 of 238 genes belonging to the NABA_ECM_REGULATORS gene set were expressed in COVID-19 PBMC and presented below as a heatmap, with genes sorted on GSEA rank. Type = Sample biotype; HC = healthy control; COVID-19 = COVID-19 patients. Condition = sample condition; HC= healthy control; ~~w~~Ward= ward patient; ICU = intensive care unit patient.


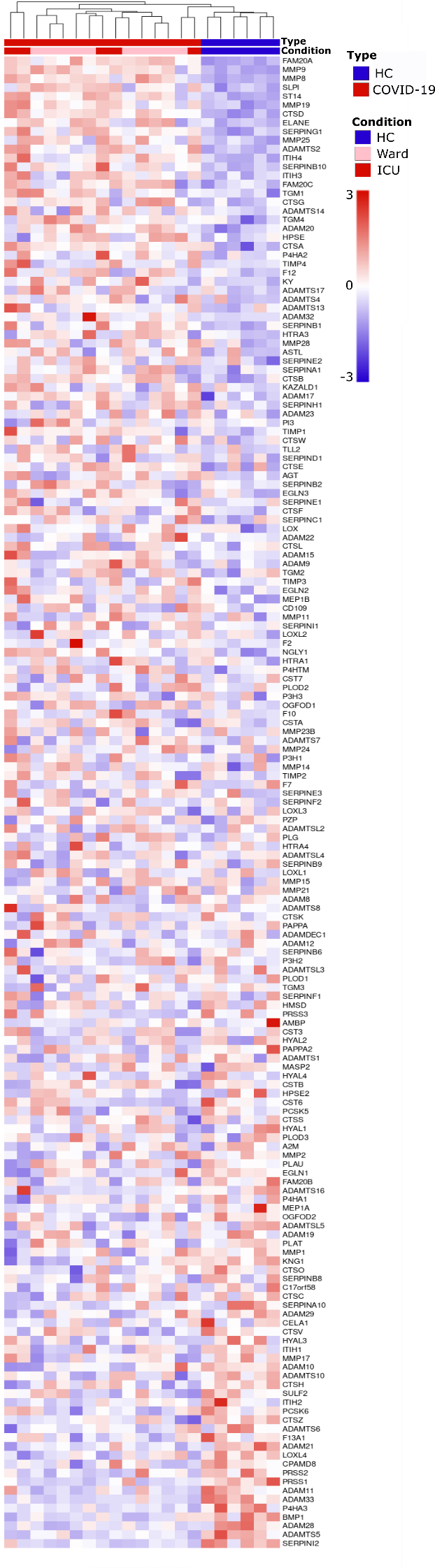


**Supplemental table S4**. **ECM Regulator gene expression in severe COVID-19.**

ECM regulator genes were significantly expressed in ICU patients compared to Ward COVID-19 patients.

| GENE | DESCRIPTION | Mean | Log2FC* | p-value* | Padj* |
| --- | --- | --- | --- | --- | --- |
| A2M | alpha-2-macroglobulin | 20,0 | -0,081 | 0,000 | 0,043 |
| ADAM15 | ADAM metallopeptidase domain 15 | 1153,0 | 0,136 | 0,018 | 0,298 |
| ADAMTS13 | ADAM metallopeptidase with thrombospondin type 1 motif 13 | 63,9 | 0,118 | 0,016 | 0,279 |
| ADAMTS2 | ADAM metallopeptidase with thrombospondin type 1 motif 2 | 1043,5 | 2,373 | 0,000 | 0,051 |
| ADAMTSL4 | ADAMTS like 4 | 892,5 | 0,093 | 0,015 | 0,271 |
| AGT | angiotensinogen | 52,0 | 0,501 | 0,003 | 0,137 |
| CPAMD8 | C3 and PZP like alpha-2-macroglobulin domain containing 8 | 175,8 | 0,088 | 0,021 | 0,317 |
| CTSA | cathepsin A | 7264,0 | 0,175 | 0,026 | 0,402 |
| CTSD | cathepsin D | 12382,6 | 0,331 | 0,010 | 0,249 |
| EGLN2 | egl-9 family hypoxia inducible factor 2 | 1834,8 | 0,142 | 0,039 | 0,421 |
| HTRA1 | HtrA serine peptidase 1 | 50,4 | 0,063 | 0,024 | 0,340 |
| ITIH3 | inter-alpha-trypsin inhibitor heavy chain 3 | 15,6 | 0,061 | 0,026 | 0,351 |
| ITIH4 | inter-alpha-trypsin inhibitor heavy chain 4 | 476,1 | 0,164 | 0,031 | 0,376 |
| LOXL1 | lysyl oxidase like 1 | 19,4 | -0,045 | 0,039 | 0,415 |
| MMP19 | matrix metallopeptidase 19 | 66,8 | 1,394 | 0,000 | 0,039 |
| MMP23B | matrix metallopeptidase 23B | 216,3 | 0,170 | 0,018 | 0,300 |
| MMP25 | matrix metallopeptidase 25 | 1845,7 | 0,960 | 0,001 | 0,078 |
| P4HA2 | prolyl 4-hydroxylase subunit alpha 2 | 8,9 | 0,035 | 0,014 | 0,269 |
| P4HTM | prolyl 4-hydroxylase, transmembrane | 875,6 | -0,300 | 0,004 | 0,153 |
| PAPPA | pappalysin 1 | 9,7 | -0,023 | 0,008 | 0,215 |
| PCSK6 | proprotein convertase subtilisin/kexin type 6 | 860,0 | 0,620 | 0,003 | 0,121 |
| PLAT | plasminogen activator, tissue type | 11,4 | -0,062 | 0,012 | 0,250 |
| PLOD1 | procollagen-lysine,2-oxoglutarate 5-dioxygenase 1 | 846,4 | 0,141 | 0,045 | 0,442 |
| SERPINB1 | serpin family B member 1 | 3300,0 | 0,218 | 0,018 | 0,344 |
| SERPING1 | serpin family G member 1 | 559,4 | 0,044 | 0,013 | 0,260 |
| ST14 | ST14 transmembrane serine protease matriptase | 751,4 | 0,113 | 0,032 | 0,382 |
| TIMP1 | TIMP metallopeptidase inhibitor 1 | 3270,5 | 0,331 | 0,010 | 0,261 |
| TIMP3 | TIMP metallopeptidase inhibitor 3 | 19,7 | 0,040 | 0,023 | 0,326 |
| TIMP4 | TIMP metallopeptidase inhibitor 4 | 7,8 | 3,325 | 0,000 | 0,001 |

* P-values, adjusted p-values (padj) and Log2fold change (Log2FC) are represented as ICU/Ward comparison values. Mean = mean expression value.

**Supplemental Table S5. ECM regulator proteins.**

Peripheral blood mononuclear cell proteins from severe COVID-19 patients that were recognised as ECM Regulator proteins.

| UniProt | SYMBOL | Log2FC* | p-val* | p-adj* |
| --- | --- | --- | --- | --- |
| P10619 | CTSA | 0,43 | 0,000 | 0,021 |
| O14672 | ADAM10 | 0,28 | 0,004 | 0,080 |
| P00488 | F13A1 | 0,67 | 0,005 | 0,088 |
| P07339 | CTSD | 0,30 | 0,007 | 0,102 |
| P50452 | SERPINB8 | -0,23 | 0,027 | 0,163 |
| P50453 | SERPINB9 | -0,21 | 0,057 | 0,236 |
| P25774 | CTSS | -0,18 | 0,163 | 0,409 |
| P53634 | CTSC | -0,19 | 0,163 | 0,410 |
| P04080 | CSTB | -0,12 | 0,253 | 0,520 |
| P07858 | CTSB | -0,10 | 0,357 | 0,618 |
| P30740 | SERPINB1 | 0,07 | 0,589 | 0,784 |
| P22894 | MMP8 | -0,17 | 0,597 | 0,787 |
| P48595 | SERPINB10 | -0,06 | 0,735 | 0,874 |
| P14780 | MMP9 | -0,11 | 0,750 | 0,883 |
| P08246 | ELANE | 0,08 | 0,762 | 0,885 |
| Q9UBR2 | CTSZ | 0,04 | 0,776 | 0,893 |
| Q9Y251 | HPSE | 0,03 | 0,884 | 0,949 |
| P35237 | SERPINB6 | -0,01 | 0,927 | 0,971 |
| P01009 | SERPINA1 | -0,01 | 0,963 | 0,987 |
| P08311 | CTSG | 0,00 | 0,998 | 0,999 |

*log2fold change (log2FC), p-value (p-val) and p-adjusted value (p-adj) are derived from *Limma* results and presented as ICU/Ward comparison values.

**Supplemental Figure S6. Regulation of ECM-related gene sets in inflamed tissue.**


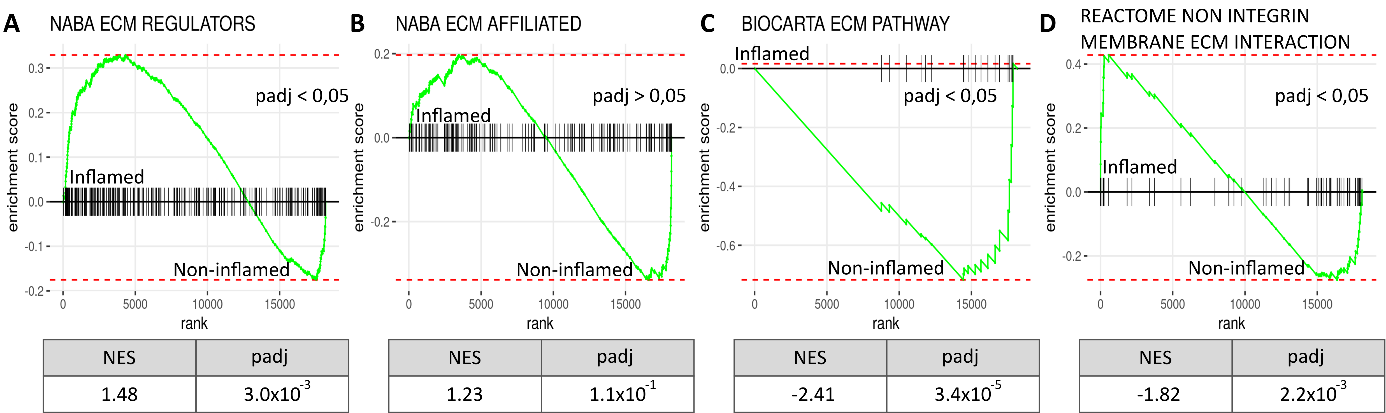


Comparing a publicly available gene expression data set of inflamed lung tissue to paired non-inflamed tissue from the same deceased COVID-19 patients (N=5, inflamed biopsies = 49, non-inflamed biopsies=17) shows ECM regulatory activity when using GSEA analysis on the gene dataset: NABA ECM REGULATORS (**A**), NABA ECM AFFILIATED (**B)**, BIOCARTA ECM PATHWAY (**C)** and REACTOME NON INTERGRIN MEMBRANE ECM INTERACTION (**D)**. The ECM regulators gene set was significantly increased in inflamed tissue. Meanwhile, the Biocarta ECM pathway and Reactome non-integrin membrane ECM interaction was significantly decreased. Finally, the ECM-affiliated gene set was not considerably regulated between the two conditions.
